# Nationwide seroprevalence of SARS-CoV-2 during the COVID-19 pandemic and prepandemic periods in Lao PDR

**DOI:** 10.1371/journal.pone.0336239

**Published:** 2025-11-13

**Authors:** Koukeo Phommasone, Chanthala Souksakhone, Anisone Chanthongthip, Ooyanong Phonemixay, Chirapha Keokhamphoui, Stéphane Priet, Karine Barthélémy, Xavier de Lamballerie, Manivanh Vongsouvath, Elizabeth A. Ashley, Audrey Dubot-Pérès

**Affiliations:** 1 Lao-Oxford-Mahosot Hospital-Wellcome Trust Research Unit, Microbiology Laboratory, Mahosot Hospital, Vientiane, Lao PDR; 2 National Blood Transfusion Center, Lao Red Cross, Rue Phai Nam, Vientiane, Lao PDR; 3 Unité des Virus Émergents (UVE: Aix-Marseille Univ, Università di Corsica, IRD 190, Inserm, IRBA), Marseille, France; 4 Centre for Tropical Medicine and Global Health, Nuffield Department of Clinical Medicine, University of Oxford, Oxford, United Kingdom; Yamagata University Faculty of Medicine: Yamagata Daigaku Igakubu Daigakuin Igakukei Kenkyuka, JAPAN

## Abstract

**Background:**

Lao PDR reported a low number of confirmed SARS-CoV-2 cases until the introduction of COVID-19 vaccines in April 2021. This raised doubts about whether the reported number reflected the true situation and questioned about possible pre-existing immunity against SARS-CoV-2.

**Methods:**

We retrospectively analyzed leftover blood donor samples collected in 2003–2004, 2015, and 2018 in Vientiane Capital, as well as samples collected in 2020 and between February and August 2021 across 18 provinces in Lao PDR to better understand the epidemiology of the infection. The samples were first screened using an anti-SARS-CoV-2 IgG ELISA (Euroimmun). The presence of anti-SARS-CoV-2 antibodies was then confirmed or refuted by testing positive and equivocal samples using anti-SARS-CoV-2 IgG CLIA (Beckman Coulter) targeting anti-Spike antibodies and SARS-CoV-2 virus neutralization test.

**Results:**

All pre-pandemic sera from Vientiane capital (79 in 2003−2004, 406 in 2015 and 191 in 2018), and all 2,225 sera collected in 2020 from eight provinces, tested negative. A total of 3,663 sera were collected prospectively in 2021 from 14 provinces. Bokeo and Oudomxay had the highest prevalence, each at 19.8%, followed by Luang Namtha at 16.1% and Salavan at 15.5%. However, the majority of samples collected from those four provinces were obtained after the outbreak surge and the introduction of COVID-19 vaccines in mid-April.

**Conclusion:**

There was no evidence of cross-reactive neutralizing antibodies prior to the COVID-19 pandemic and the seroprevalence of SARS-CoV-2 was confirmed to be low in Lao PDR before the introduction of COVID-19 vaccines and the surge in infections from May 2021.

## Introduction

Coronavirus disease 2019 (COVID-19), was confirmed to be caused by SARS-CoV2, a novel coronavirus, by the Chinese authorities on 7 January 2020, following a cluster of patients with pneumonia of unknown aetiology linked to the seafood market in Wuhan City, Hubei Province of China in December 2019 [1]. Due to the rapid spread across the globe, the declaration of a COVID-19 pandemic was made in March 2020 by World Health Organization (WHO).

Transmission of SARS-CoV2 in Southeast Asian countries was not as widespread in 2020 as in other parts of the world despite close proximity to China [2]. This was attributed to early border closure at the end of January and early February 2020 [3]. In Laos, where public health infrastructure is limited, issuance of visas to travelers from China was suspended starting on February 2, 2020 [4], followed by restriction on all international travel beginning on March 29, 2020 [5]. Other non-pharmaceutical interventions, such as mask-wearing, were implemented in early February 2020, followed by school and workplace closures, as well as the prohibition of mass gatherings, in March 2020 [5]. Lao PDR was not only the last country in the region to report its first confirmed COVID-19 cases on March 24, 2020 [6], but it also had the lowest number of COVID-19 cases compared to neighbouring countries such as Cambodia, Thailand, Vietnam, and Myanmar [3]. Following the first confirmed case, the number of confirmed cases remained low until the end of April 2021, after which it gradually increased and surged at the end of August 2021 [5]. Mass vaccination was first administered to frontline workers and healthcare workers in March 2021, followed by the general public in April 2021 using Sinopharm COVID-19 vaccine from China and AstraZeneca from the COVAX facility [7,8].

In Laos, the reported number of COVID-19 cases was primarily based on testing symptomatic individuals, which may have overlooked asymptomatic cases. Estimates of asymptomatic infections from other countries varied from less than one percent to over 50% [9]. He et al. found that asymptomatic rate could be up to 80% in Wuhan, China [10]. Underestimation of the true number of infections can also result from availability, accessibility, sensitivity and specificity of diagnostic tests in use. In Laos, RT-PCR was initially only available in the capital city, Vientiane. By September 2020, ten provincial laboratories had the capacity to test for COVID-19 using GeneXpert machines with plans to expand testing to the remaining provinces thereafter [11].

Although viral RNA detection by real-time RT-PCR is the most reliable technique to confirm infections or incidences, it detects active or current infection only. In contrast, antibody tests can indicate past exposure to the infection, even in asymptomatic individuals who might have been missed by PCR testing or those who recovered a long time ago. Therefore, a better estimation of the prevalence of SARS-CoV-2 infections can be achieved using serological tests, which help to understand the distribution and level of SARS-CoV-2 infection [12].

The first extensive seroprevalence study in Laos, conducted between August and September 2020, included community members and healthcare workers across five provinces, including Vientiane Capital, as well as individuals with wildlife contacts in Vientiane province. This study found no evidence of significant SARS-CoV-2 circulation [13].

To provide more data to estimate the nationwide prevalence of SARS-CoV2 in Laos before and at the early stage of the pandemic, we conducted a seroprevalence study of SARS-CoV2 in blood donors from each province across the country by detecting anti-SARS-CoV-2 neutralizing antibodies (NAb) in stored blood donor samples collected during the pre-pandemic and pandemic periods.

## Materials and methods

### Samples

Blood was collected by the Lao Red Cross National Blood Transfusion Center (NBTC), Provincial Blood Centres and some district hospitals performing blood donation. In routine practice, 5 mL of blood from each donation was centrifuged then the serum was aliquoted. One mL from leftover serum after NBTC routine screening procedure was used for this study. In each province, blood donors were recruited either at the Provincial Blood Centers or through mobile blood donation units that visited various locations, such as schools or workplaces, within the province. Serum aliquots were frozen at −20°C on collection sites then sent to NBCT in Vientiane capital, then sent to LOMWRU to be stored at −80°C until used for laboratory assay.

We tested stored blood donor samples collected in 2003, 2004, 2015, 2018 and 2020, as well as prospectively collected samples between February and August 2021. All samples became accessible for this study soon after we received ethical approval on 21/08/2021.

We expected to collect prospectively 300 samples per province for the samples collected between February and August 2021. The sample size calculation for each province was based on an assumed prevalence of 25% of people having antibodies against SARS-CoV-2, with a 95% confidence level and a margin of error of 5%.

### Ethics

The use of blood donor samples collected in 2003, 2004 was first approved by National Ethics Committee for Health Research (No:133/NECHR) on 23/07/2007. An extension was later granted to store and include samples collected in 2015 and 2018 (approval No: 05/NECHR, dated 17/1/2019) for use as control samples in studies conducted in Laos. This study was subsequently approved by the Lao University of Health Science Research Ethics Committee on 11 February 2021, and an amendment was later approved (No. 241, dated 21/08/2021) and by the Oxford Tropical Research Ethics Committee (OxTREC reference: 52−20) for the use of previously stored blood samples as well as samples collected from blood donors in 2020 and 2021. The donors gave written consent for their leftover blood to be used for research purposes at the time of donation.

### Laboratory assays

All sera were tested at Laos-Oxford-Mahosot Hospital- Wellcome Trust Research Unit, Vientiane Capital, Lao PDR, by anti-SARS-CoV-2 IgG ELISA (Euroimmun) targeting anti-Spike (S1) antibodies, following manufacturer’s instructions. Positive and equivocal samples were then sent and tested at the Emerging Virus Unit, Aix Marseille University, France, by anti-SARS-CoV-2 IgG CLIA targeting anti-Spike (receptor binding domain, RBD) antibodies (Beckman Coulter), following manufacturer’s instructions, and virus neutralization test (VNT) performed in a 96-well format, using SARS-CoV-2 (BavPat1) as previously described [14]. VNT was performed in duplicate and considered as positive if both gave a titre ≥40.

### Result interpretation

The Euroimmun anti-S1 IgG ELISA was used as a screening test targeting the broader S1 region of the SARS-CoV-2 spike protein. False-positive results occasionally occur with ELISA, likely due to cross-reactivity and the limited specificity of the IgG ELISA technique. To improve the specificity of the results, we subsequently used CLIA methods targeting the conserved RBD region, along with a neutralization assay, which is considered the gold standard for serology.

Therefore, a sample was confirmed for the presence of anti-SARS-CoV-2 antibodies if it was positive with at least two of the VNT, ELISA or CLIA tests.

### Data analysis and map production

Data were entered and cleaned using Microsoft Excel. Descriptive statistics were used to summarize demographics of the study population. Age was presented as the median with interquartile range (IQR). Categorical variables such as sex and serological results were summarized using frequency and percentage. A seroprevalence map was produced using QGIS (Quantum Geographic Information System) version 3.40.5. The administrative boundary shapefile was obtained from the GADM database of Global Administrative Areas and used for non-commercial academic purposes under GADM license.

## Results

### Characteristics of the participants

Retrospective samples from blood donors in Vientiane capital from the pre-pandemic period were analyzed: 36 sera from 2003, 43 sera from 2004, 406 from 2015, and 191 from 2018 with male to female sex ratios of 3.8, 1.6, and 1.5, respectively, and median ages (IQR) of 20 years (18 –28 ), 29 years (23 –35), and 31years (26 –37), respectively (**Table 1**). From the pandemic period, 2295 samples collected from January to December 2020 from 8 provinces were also analyzed. These samples had a male to female ratio of 2.5 and a median age of 27 years (IQR:19–33). Additionally, 3663 prospective samples were collected from 14 provinces from February to August 2021, with a male to female ratio of 1.4 and a median age of 30 years (IQR:24–37). For provinces Khammoune, Phongsaly, Savannakhet and Xekong, only 3, 12, 6 and 2 samples were collected in 2021 respectively and were not included in the analysis.

**Table 1:** Characteristics of blood donors.

COVID19 pre-pandemic period									
	2003-2004			2015			2018		
Province	n	Sex ratio (M/F)	Age (y), median (IQR)	n	Sex ratio (M/F)	Age (y), median (IQR)	n	Sex ratio (M/F)	Age (y), median (IQR)
Vientiane Capital	79	3.8 (61/16)	20 (18 –28)	406	1.6 (246/154)	29 (23 –35)	191	1.5 (116/75)	31 (26 –37)
**COVID19 pandemic period**									
	**2020**			**2021**					
**Province**	**n**	**Sex ratio (M/F)**	**Age (y), median (IQR)**	**n**	**Sex ratio (M/F)**	**Age (y), median (IQR)**			
Vientiane Capital	303	2.5 (216/87)	27 (19 –33)	328	1.4 (191/137)	30 (24 –37)			
Attapue	171	2.2 (118/53)	19 (18 –20)	283	1.7 (179/104)	20 (18 –30)			
Bolikhamxay	296	1.5 (179/117)	18 (17 –20)	289	2.0 (194/95)	19 (18 –26)			
Bokeo				293	4.1 (235/58)	27 (22 –33)			
Champasak				296	2.0 (194/98)	20 (18 –24)			
Houaphanh	286	1.6 (177/109)	18 (17 –19)	282	2.1 (192/90)	21 (18 –31)			
Luang-Namtha	354	2.9 (264/90)	25 (19 –33)	143	2.3 (99/44)	29 (24 –35)			
Luang-Prabang	291	1.0 (148/143)	29 (23 –36)	303	1.2 (164/139)	25 (19 –34)			
Oudomxay				253	2.1 (172/81)	29 (23 –35)			
Salavan				238	2.5 (170/68)	26 (20 –33)			
Vientiane Province				282	2.6 (203/79)	23 (18 –31)			
Xayabury	305	2.2 (209/96)	25 (19 –32)	291	2.5 (208/83)	26 (19 –32)			
Xaysomboune				88	7 (77/11)	21 (19-24.5)			
Xiengkhuang	289	172/117	20 (18 –27)	294	2.8 (217/77)	19 (18 –24)			

### SARS-CoV-2 serology results

Leftover blood donor samples collected prior to the COVID-19 pandemic, as well as all samples collected in 2020, tested negative for anti-SARS-CoV-2 antibodies.

**Fig 1:**
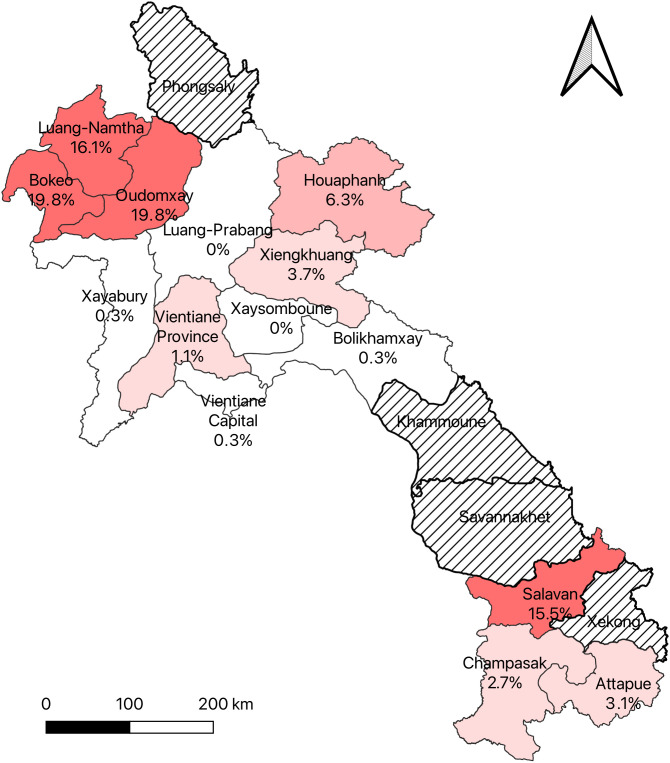
Map of Lao PDR showing seroprevalence of SARS-CoV-2 between Feb. and Aug. 2021. Striped provinces: No data available.

**Fig 2:**
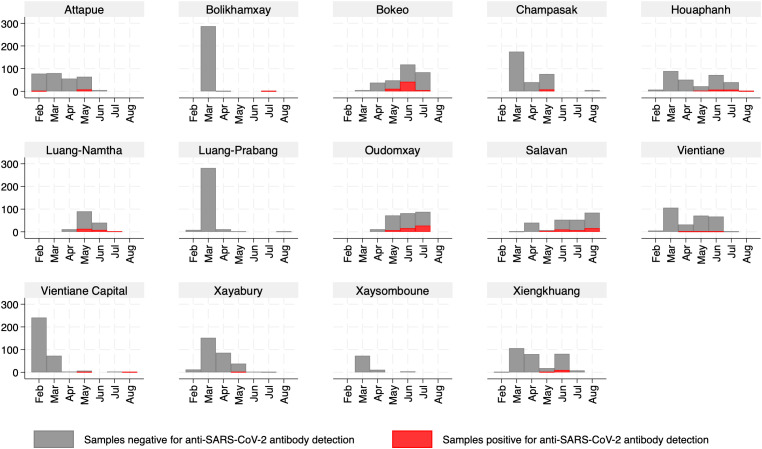
The distribution of samples collected between February and August 2021 in each province and the number of participants positive for antibodies against SARS-CoV-2. Footnote: Anti-SARS-CoV-2 antibodies were considered positive if at least two of the following tests were positive: VNT, ELISA and CLIA tests.

For samples collected in 2021, 11.6% (429/3,663) tested positive by ELISA, ranging from 0 in Xaysomboune to 36.1% (80/238) in Salavan. There were 429 samples that tested positive or equivocal by ELISA and were submitted to confirmatory testing. Anti-SARS-CoV-2 antibodies were confirmed in 6.0% (220/3,663) of participants. The seroprevalence of SARS-CoV-2 was highest in Bokeo and Oudomxay, each at 19.8%, followed by Luang-Namtha at 16.1% and Salavan at 15.5%. There were no positive samples in Luang-prabang and Xaysomboune, and only 0.3% in Vientiane Capital (**Fig 1 and Table 2**). The majority of positive samples were collected between May and August 2021 with only one positive in February from Attapue and one in April from Vientiane province. Nearly all samples from Luang-Prabang, Vientiane Capital, Xaysomboune, Bolikhamxay, Xayabory and Champasack were collected before May 2021, and all were negative (**Fig 2**).

**Table 2:** The SARS-CoV-2 antibody ELISA, confirmatory test results and estimated SARS-CoV-2 seroprevalence in blood donor participants for each province of Lao PDR.

Pre COVID19 pandemic period												
	2003-2004				2015				2018			
Province	Total n	Non neg by ELISA		SERO	Total n	Non neg by ELISA		SERO	Total n	Non neg by ELISA		SERO
		n (%)	Conf (%)			n (%)	Conf (%)			n (%)	Conf (%)	
Vientiane Capital	79	0		0	406	2 (0.6%)	0/2	0	191	0		0
**COVID19 pandemic period**												
	**2020**				**2021**							
**Province**	**Total n**	**Non neg by ELISA**		**SERO**	**Total n**	**Non neg by ELISA**		**SERO**				
		**n (%)**	**Conf (%)**			**n (%)**	**Conf (%)**					
Vientiane Capital	303	17 (5.6)	0/17 (0)	0	328	5 (1.5)	1/4* (25.0)	0.3% (1/328)				
Attapue	171	2 (1.7)	0/2 (0)	0	283	12 (4.0)	9/12 (75.0)	3.2% (9/283)				
Bolikhamxay	296	12 (4.1)	0/11*(0)	0	289	1 (0.3)	1/1 (100.0)	0.4% (1/289)				
Borkeo					293	89 (30.4)	58/84* (69.0)	19.8% (58/293)				
Champasak					296	21 (7.1)	8/20* (40.0)	2.7% (8/296)				
Houaphanh	286	6 (2.1)	0/5*(0)	0	282	36 (12.5)	18/36° (50.0)	6.4% (18/282)				
Luang-Namtha	354	4 (1.1)	0/4 (0)	0	143	46 (32.2)	23/45* (51.1)	16.1% (23/143)				
Luang-Prabang	291	5 (1.7)	0/5 (0)	0	303	5 (1.7)	0/3*^+^(0)	0				
Oudomxay					253	90 (35.6)	50/89* (56.2)	19.8% (50/253)				
Salavan					238	86 (36.1)	37/66*^&^ (56.1)	15.5% (37/238)				
Vientiane Province					282	9 (3.2)	3/8* (37.5)	1.1% (3/282)				
Xayabury	305	10 (3.3)	0/8* (0)	0	291	7 (2.4)	1/6* (16.7)	0.3% (1/291)				
Xaysomboune					88	0		0				
Xiengkhuang	289	1 (0.3)	0/1 (0)	0	294	22 (7.5)	11/22 (50.0)	3.7% (11/294)				
Total	2295	57 (2.5)	0/53* (0)	0	3663	429 (11.6)	220/396* (55.3)	6.0% (220/3663)				

Non-neg by ELISA: n = number of samples found positive or equivocal by anti-SARS-CoV-2 IgG ELISA (Euroimmun). Conf= among the non-negative samples by ELISA, the number of samples for which the detection of anti-SARS-CoV-2 antibodies was confirmed (at least two of ELISA, CLIA or VNT were positive).

* samples not tested for at least one of the two confirmatory tests due to volume not available.

° two samples with inconclusive results, ^+^ one sample with inconclusive results, ^&^ two samples with inconclusive results

SERO: SARS-CoV-2 seroprevalence = proportion of samples confirmed for anti-SARS-CoV-2 antibodies over the total number of samples tested.

Details of test results for the 488 non-negative samples by ELISA are provided in S1 Table and the population size in Laos in 2021 in S2 Table.

Among 2,252 samples collected between February and April 2021, only two (0.08%) tested positive. By comparison, 218 out of 1,412 samples (15.4%) collected between May and August 2021 tested positive.

## Discussion

Laos was considered vulnerable to COVID-19 due to its shared border with China, where SARS-CoV-2 was first reported. However, similar to other ASEAN countries, the number of reported cases was relatively low throughout 2020 and into 2021 [2]. A systematic review and meta-analysis on the global seroprevalence of SARS-CoV-2 antibodies in 2020 showed that seroprevalence in the general population was low, with a median of 4.5% (IQR: 2.4–8.4%), and could be as high as 59% among persons working in care facilities. The study also found that Southeast Asia had the lowest seroprevalence at 0.6%, compared to 19.5% in Sub-Saharan Africa [15]. Another repeated study, which collected data between January 2020 and May 2022, showed that by September 2021, the global SARS-CoV-2 seroprevalence increased to 59.2% (95% CI: 56.1%–62.2%). The study also reported a steep increase in seroprevalence in Africa, rising from 26.6% in early 2021 to 88.5% by the end of 2021 [16]. From the first reported case in March 2020 up to the first two months of our prospective sampling (February-March 2021), only 49 confirmed cases were reported [17]. Our study confirmed low level of community transmission of COVID-19 throughout Laos before May 2021 with only two out of 2251(0.09%) samples collected testing positive. Subsequently, there was noticeable rise in the seroprevalence among blood donors, with 218 out of 1,412 (15.4%) samples testing positive. This increase corresponded with the rise in reported cases, from 49 cases in March 2021–555 cases by the end of April and 15,095 cases by the end of August 2021 [5,18]. Our findings are in accordance with the first seroprevalence survey conducted by Virachith and colleagues between August and September 2020 in community and high-risk groups in Vientiane capital, Luang-Prabang, Oudomxay, Savannakhet and Champasak provinces, confirming that the community transmission was low until September 2020 [13]. They found that 5.2% (131/2433) and 2.1% (19/1061) of the general population tested positive for anti-N antibodies and anti-S antibodies tested by ELISA, respectively; 2% (13/666) and 1.4% (4/282) of healthcare workers; and 20.3% (15/74) and 6.8% (5/74) of individuals with bat/wildlife contact. However, only 0.1% (2/3173) of participants were positive for both antibodies and the authors believe that the seropositivity observed in their study was due to cross-reactivity with non-SARS-CoV-2 coronaviruses.

We found the highest prevalence in three northern provinces (Bokeo, Luang-Namtha and Oudomxay) and one southern province (Salavan) of Laos, but all of the samples positive for anti-SARS-CoV-2 antibodies were collected after April 2021, when COVID-19 vaccines had already been rolled out and the number of clinical cases was beginning to increase. During the study period, the highest number of reported COVID-19 cases, according to the government, was in Savannakhet, followed by Champasack, Khammuan, and Vientiane Capital [5]. Unfortunately, the number of blood donors sampled from Khammuan, Savannakhet, Phongsaly, and Sekong was limited, preventing us from obtaining SARS-CoV-2 seroprevalence data for these provinces. The low number of positive samples in Vientiane Capital and Champasack Province in our study may be attributed to the fact that most blood donations were collected before May, a period of low transmission.

There was speculation that the actual case numbers might have been underestimated, potentially due to low testing rates or to pre-existing immunity generated by exposure to other related coronavirus circulating in the area prior to the pandemic [19]. However, analysis of 676 samples collected between 2003 and 2018 revealed no detection of SARS-CoV-2 neutralizing antibodies.

The COVID-19 situation report at the end of May 2021 indicated that Huaphan, Sekong, Attapeu, and Xaisomboun were still in Stage 0, with no reported cases in these four provinces [5]. However, in Attapeu, one out of 78 blood donor samples collected in February and eight out of 64 samples collected in May tested positive for antibodies against SARS-CoV-2. Additionally, three out of 22 blood donor samples collected in May in Houaphanh were positive, suggesting some silent circulation of SARS-CoV-2 in these two provinces despite the absence of confirmed cases.

Using blood donors for a seroprevalence study was very convenient for identifying infection hotspots. However, there are several limitations to using blood donors, such as selection bias, age and gender distribution, and geographical bias. In our study, the most significant limitation was the timing of blood donations, as the samples collected were not equally distributed over the study period in each province. For example, our study was conducted prospectively between February and August 2021; some provinces sent nearly all their samples collected before May 2021, while others sent samples collected after that, which distort the actual epidemiology of the infection.

## In conclusion

Community transmission of SARS-CoV-2 in Laos before May 2021 was low all over the country, which was consistent with government reports. This was likely due to strict control measures implemented. The high prevalence in certain provinces also aligned with the reported increase in cases and the rollout of the COVID-19 vaccine.

## Supporting information

S1 Table ELISA test results for non-negative samples. (XLSX)

S2 Table Population in Laos in 2021. (XLSX)
